# Solution-Mediated Modulation of *Pseudomonas aeruginosa* Biofilm Formation by a Cationic Synthetic Polymer

**DOI:** 10.3390/antibiotics8020061

**Published:** 2019-05-10

**Authors:** Leanna L. Foster, Shin-ichi Yusa, Kenichi Kuroda

**Affiliations:** 1 Macromolecular Science and Engineering Program, University of Michigan, Ann Arbor, MI 48109, USA; llfoster@umich.edu; 2 Department of Applied Chemistry, University of Hyogo, 2167 Shosha, Himeji, Hyogo 671-2280, Japan; yusa@eng.u-hyogo.ac.jp; 3 Department of Biologic and Materials Sciences & Prosthodontics, School of Dentistry, University of Michigan, Ann Arbor, MI 48109, USA

**Keywords:** biofilms, antimicrobial polymers, materials

## Abstract

Bacterial biofilms and their associated infections are a continuing problem in the healthcare community. Previous approaches utilizing anti-biofilm coatings suffer from short lifetimes, and their applications are limited to surfaces. In this research, we explored a new approach to biofilm prevention based on the hypothesis that changing planktonic bacteria behavior to result in sub-optimal biofilm formation. The behavior of planktonic *Pseudomonas aeruginosa* exposed to a cationic polymer was characterized for changes in growth behavior and aggregation behavior, and linked to resulting *P. aeruginosa* biofilm formation, biomass, viability, and metabolic activity. The incubation of *P. aeruginosa* planktonic bacteria with a cationic polymer resulted in the aggregation of planktonic bacteria, and a reduction in biofilm development. We propose that cationic polymers may sequester planktonic bacteria away from surfaces, thereby preventing their attachment and suppressing biofilm formation.

## 1. Introduction

Synthetic surfaces of medical devices and implants are susceptible to microbial colonization and biofilm formation [1,2,3,4,5], which contribute to at least 60% of healthcare-acquired infections [6]. Biofilms are difficult to treat by antibiotics, with research citing poor antibiotic penetration, high bacteria density, reduced metabolic activity, and slow growth rate [7,8,9,10]. These factors can result in sub-lethal antibiotic exposure and contribute to resistance development [7,11]. Additionally, biofilms are physically robust, and difficult to remove mechanically. Specifically, *Pseudomonas aeruginosa* frequently develops into biofilms and consequently chronic infections of the respiratory system, especially in immunocompromised patients [12,13,14]. The prevention of biofilm formation is a primary challenge in modern biomaterials science in order to prevent chronic infections. It is, therefore, critically important to create effective anti-biofilm strategies.

The long-term goal of this study is to develop a new effective method to address the challenge of *P. aeruginosa* biofilm-associated infections. The main-stream approach in the general area is to modify device surfaces with antibacterial or anti-fouling polymers, which kill bacteria on contact or effectively repel them [15,16,17,18,19,20,21,22,23]. However, while some recent approaches show promising results, it has been long-term scientific and technical challenges to generate surface materials that can inherently overcome the sophisticated biological adhesion mechanisms of bacteria as well as meet requirements for use in products in terms of efficacy and manufacturing. In practice, once exposed to physiological conditions, modified surfaces suffer from a short lifetime due to protein and cell accumulations, while regenerative surfaces are of recent interest in the field [24,25]. In addition, most approaches require surface chemistries on existing products, which are generally chemically inert, and some products are not tolerant to chemical exposures. These problems lead us to explore a new bacterial target and an alternative approach to regulate biofilm formation. To that end, we hypothesize that the surface properties of planktonic bacteria in solution can be targeted to modulate bacterial aggregation/adhesion and thus prevent biofilm formation. More specifically, the biofilm formation is very orchestrated, involving changes in gene expression, spatial arrangement of bacteria, and extracellular biopolymers, cell-cell communications, and timings of these events [26]. Therefore, disrupting such natural mechanisms may cause sub-optimal changes in the biofilm development, leading to decreased biofilm formation. 

**Figure 1:**
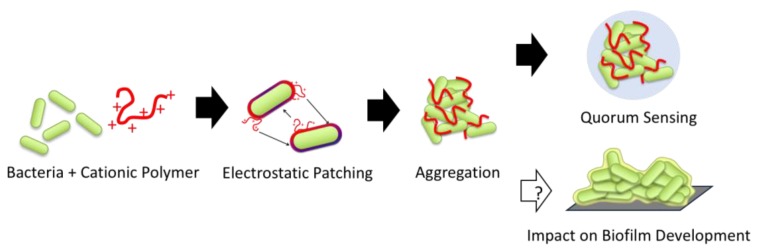
Cationic polymers can control bacteria aggregation, which has been previously linked to quorum sensing, an important part of biofilm formation. However, no studies have linked polymer modulated bacteria activity with biofilm development.

In academic research and industrial settings, many applications of polymers have been reported for their physical and biological efficacies through the direct interactions between polymers and bacteria. For example, the bacterial surface modification by cationic polymers and subsequent aggregate formation or flocculation have been utilized to separate bacteria from solution for water purification and bioreactors [27,28,29]. These polymers exploit the electrostatic interaction with negatively-charged bacterial cell surfaces, which causes charge neutralization, electrostatic patching, and polymer bridging of bacterial cells, resulting in macroscopic aggregates or precipitates [30,31,32]. However, it remains unclear if such polymer-induced aggregates favor the formation of mature biofilms or rather prevent it, while it may be intuitive that they may pre-set bacterial assembly for mature biofilm structures. Similarly, previous studies used cationic polymers as a bacteria sequestrant, which causes clustering of bacteria in solution, triggering quorum sensing cell-cell communications [33,34]. While these studies elegantly demonstrate translation of polymer-mediated cell clustering to biological response of bacteria, it is not clear if bacteria clusters in solution would contribute to biofilm formation or not [35]. There is a gap in our knowledge on how the polymer-bacteria interactions modulate biofilm formation (Figure 1). Such knowledge is critical to design and develop new anti-biofilm materials.

In this study, we take a step forward to understanding the polymer-bacteria interactions that govern biofilm formation toward the development of anti-biofilm polymers. Specifically, because of its use in many applications already, this study is a first and important step to understand how cationic polymers induce bacterial aggregates in solution and how the aggregates contribute to biofilm formation or prevention. Particularly, the purpose of this study is to provide foundational knowledge to guide future anti-biofilm approaches rather than elucidating the molecular mechanism of biofilm formation. *Pseudomonas aeruginosa* PAO1 was chosen as a model bacterium for this study because of its nature as an opportunistic pathogen, which frequently develop into biofilms and consequently chronic infections [12]. Our study demonstrates that a cationic polymer used in this study suppressed biofilm formation by sequestration of planktonic bacteria.

## 2. Results

### 2.1. Polymer Design, Synthesis, and Characterization

**Figure 2:**
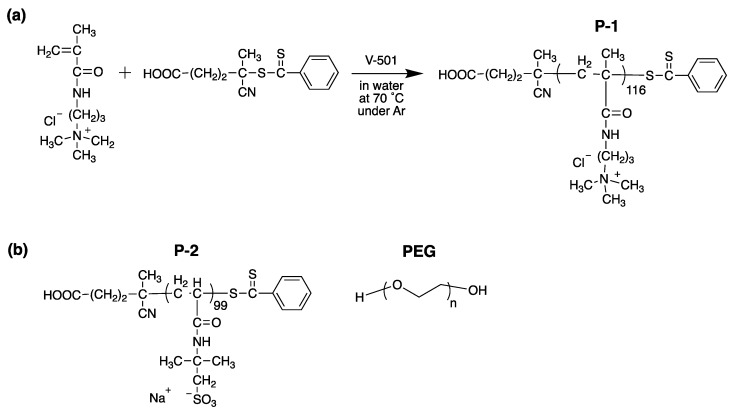
Synthesis scheme of (**a**) P-1 and (**b**) structures of P-2 and PEG.

To test the hypothesis that cationic polymers are capable of modulating biofilm formation through bacterial assembly disruption, model cationic polymer poly[(3-methacryloylamino)propyl] trimethylammonium chloride (P-1) was synthesized (Figure 2). 

The P-1 structure was chosen for this study because the quaternary ammonium groups provide permanent positive charges, while some conventional cationic polymers such as poly(ethylene imine)s contain primary ammonium groups. In contrast to primary ammoniums, quaternary ammonium groups are pH-independent, which would minimize the effect of microenvironment pH on the binding behavior of polymer chains onto the bacterial surface. As a control to determine if the observed biological effect is due to the cationic properties of P-1 or not, poly(2-acrylamido-2-methylpropane sulfonic acid) (P-2) was synthesized, which are negatively charged in a broad range of pH (Figure 2). Similar degrees of polymerization (~100) between P-1 and P-2 was selected to ensure comparable number of charges. In addition, neutral PEG will be also tested in this study. We will test the hypothesis by using these polymers with different charged groups to determine the role of charges on bacterial aggregation and biofilm formation.

P-1 and P-2 were synthesized by reversible addition-fragmentation chain transfer (RAFT) polymerization in order to achieve controlled molecular weight with narrow dispersity (*Đ*) (Figure 2). ^1^H NMR analysis confirmed the polymerization and removal of residual monomers. The degree of polymerization (DP) was determined by comparing of integral intensity ratio of peaks from the chain transfer agents to methylene and methyl in the polymer side-chains (Table 1). The number-average molecular weight (*M_n_*) was calculated using DP and molecular weights of monomer and RAFT agent. GPC was used to determine *M_n_*, molecular weight (*M_w_*), and *Đ*, which were compared to *M_n_* determined by ^1^H NMR. The molecular weights of these polymers are ~20,000 g/mol, which is relatively smaller than conventional polymers used as flocculants. Lower molecular weight polymers (~20,000 g/mol) were utilized as an initial model for this study to focus on the charge effects on bacterial aggregation and biofilm formation rather than the effect of polymer sizes and a combined effect with polymer bridging in order to elucidate a simple rule in polymer-bacteria interactions for biofilm modulation [36,37]. 

### 2.2. Planktonic Bacteria-Polymer Interactions and Consequences

#### 2.2.1. Antimicrobial Activity of Polymers

**Figure 3:**
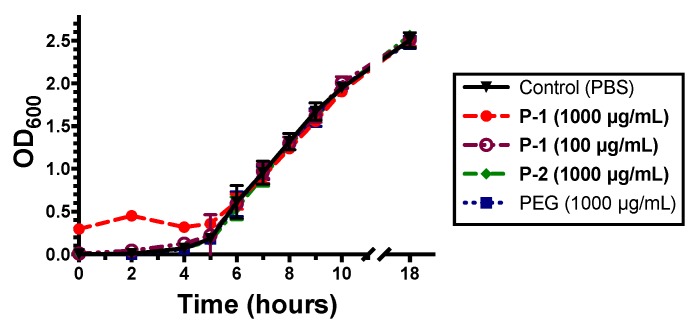
Effect of polymers on planktonic *P. aeruginosa* growth behavior in tryptic soy broth (TSB) with orbital shaking in the presence of P-1, P-2 or PEG. The data points and error bars represent the average and standard deviation from three independent experiments (*n* = 3). The data points at the time of 0 were taken immediately after the addition of the polymers.

To determine if P-1 exhibited either bacteriostatic or bactericidal effects, the growth behavior of *P. aeruginosa* was observed during log phase growth by optical density (OD_600_) (Figure 3). 

When incubated with bacteria suspensions, high P-1 concentration of 1000 µg/mL resulted in an increase in optical density during the first 4 h but did not subsequently change growth behavior. 

This increased initial OD_600_ value is likely due to aggregation of bacteria in solution resulting in greater light scattering. The P-1 induced aggregation was further explored and discussed later. Lower P-1 concentration of 100 µg/mL did not induce noticeable changes in OD_600_ and likewise did not affect bacterial proliferation (Figure 3). Neither P-2 nor PEG exhibited any changes to the bacterial growth behavior (Figure 3), suggesting no growth inhibition. After 18 h of growth, the OD_600_ of bacteria was ~2.5 for all polymers, indicating polymers had no effect on the growth. 

The antimicrobial activity of the polymers were assessed using the standard protocol to determine the minimum inhibitory concentration (MIC) [38,39], which is the concentration of polymer necessary to completely inhibit growth of bacteria during long term incubation (~18 h). The antimicrobial activity of polymers was evaluated against exponential phase *P. aeruginosa*, as well as *Escherichia coli* and *Staphylococcus aureus* (Table 2). P-1, P-2, and PEG did not show any growth inhibition effect against *P. aeruginosa, E. coli*, and *S. aureus* up to 1000 µg/mL, the highest concentration tested.

#### 2.2.2. Bacterial Aggregation and Flocculation

**Figure 4:**
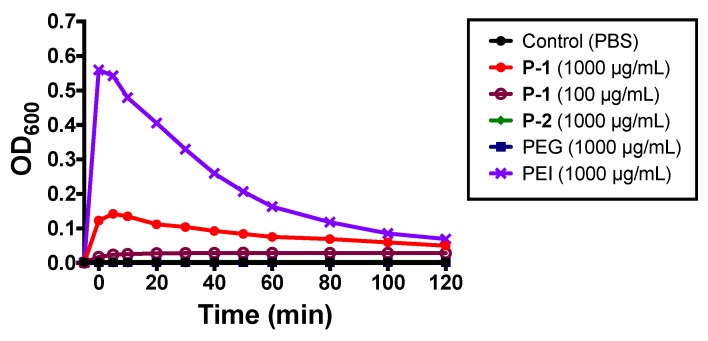
Effect of polymers on planktonic *P. aeruginosa* solution behavior. Representative data of optical density of *P. aeruginosa* in TSB incubated with P-1, P-2, PEG, or PEI at 1000 µg/mL, and PBS at room temperature without orbital shaking. The polymers were added into the bacterial suspension at time 0 min.

Aggregation/flocculation of planktonic bacteria was evaluated by changes in optical density (OD_600_) under static conditions. Specifically, flocculants increase optical density, indicating the formation of larger particles, followed by a decrease of optical density as aggregates settle to the bottom of the vessel. Branched polyethylene imide (PEI) is a known flocculant and was used as a comparison to P-1, P-2, and PEG. PEI caused a sharp increase in OD_600_, followed by a gradual reduction as aggregates precipitated to the bottom of the solution (Figure 4). 

Likewise, P-1 at 1000 µg/mL also increase and a slow reduction in OD_600_ suggesting aggregation of bacteria (Figure 4), which reflects the initial large OD value in the bacterial growth curve with P-1 (Figure 3). Lower P-1 concentration of 100 µg/mL did not induce noticeable change in OD_600_ (Figure 4). P-2 and PEG failed to induce changes in OD_600_ (Figure 4).

**Figure 5:**
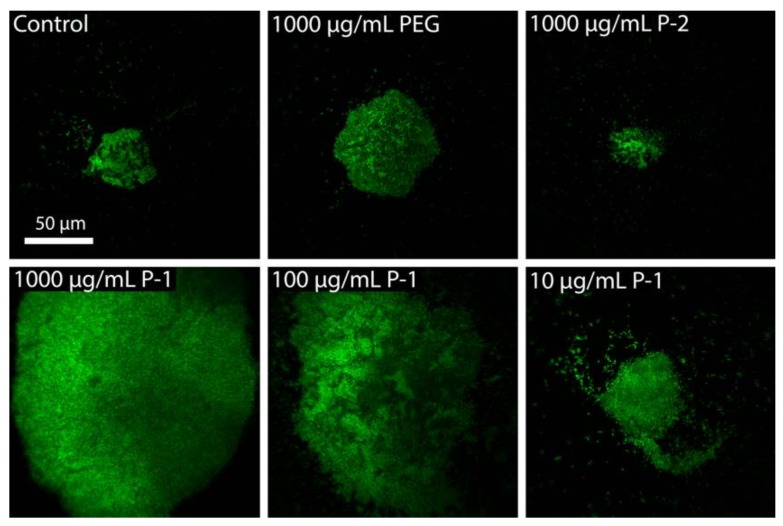
Representative images depicting aggregates of 1.0 OD_600_ solutions following 15 min incubation with polymers solutions in PBS.

The presence of aggregates was also confirmed by fluorescence microscopy. Higher bacteria density (OD_600_ = 1.0) was used to better visualize bacteria. In cultures of *P. aeruginosa* without any polymers, bacterial cells were observed in a diversity of aggregate sizes up to 50 µm diameter in addition to individual planktonic cells (Figure 5). 

This is consistent with previous reports of auto-aggregation in nutrient deficient medium, such is the case of stationary phase bacteria [40]. With the addition of PEG or P-2, the aggregates did not dissociate nor increase in size, indicating neutral or anionic charged polymers do not perturb bacteria surface charge (Figure 5). At high P-1 concentrations of 1000 and 100 µg/mL, large aggregates up to 200 µm diameter became evident in addition to smaller aggregates with 20–50 µm in diameter (Figure 5. These results are consistent with optical density aggregation behavior, in which high concentrations of P-1 increased bacterial aggregation. 

### 2.3. Development of Bacterial Biofilms

**Figure 6:**
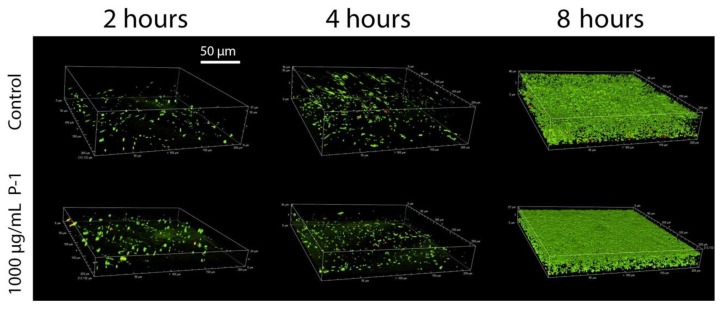
Representative three-dimensional renderings of *P. aeruginosa* biofilm development over two, four, and eight hours in the absence (control) and presence of P-1 at 1000 µg/mL.

To further test the hypothesis, the effect of *P. aeruginosa* aggregation on bacterial adhesion and biofilm formation was studied through confocal microscopy in the presence of P-1 (1000 µg/mL) while P-2 and PEG did not induce bacterial aggregation in planktonic bacteria. The formation of 3-dimensional structures can be seen observed in as little as two hours, originating from a layer of single cell attachments to the surface (Figure 6). 

After four hours, the biofilm has thickened with a dispersion of live bacteria throughout, which divide and more densely populate the biofilm after eight hours (Figure 6). In the presence of P-1, biofilms appear to be initiated by single bacterium, or small clusters of bacteria (diameter < 10 µm), but do not appear to include large aggregates that dominate the biofilm (Figure 6). After four hours, the biofilm thickens and becomes densely populated with live bacteria after eight hours, such as was seen in the case of control bacteria (Figure 6). 

### 2.4. Effect of Polymer Incubation on Biofilm Development 

#### 2.4.1. Accumulation of Total Biomass

**Figure 7:**
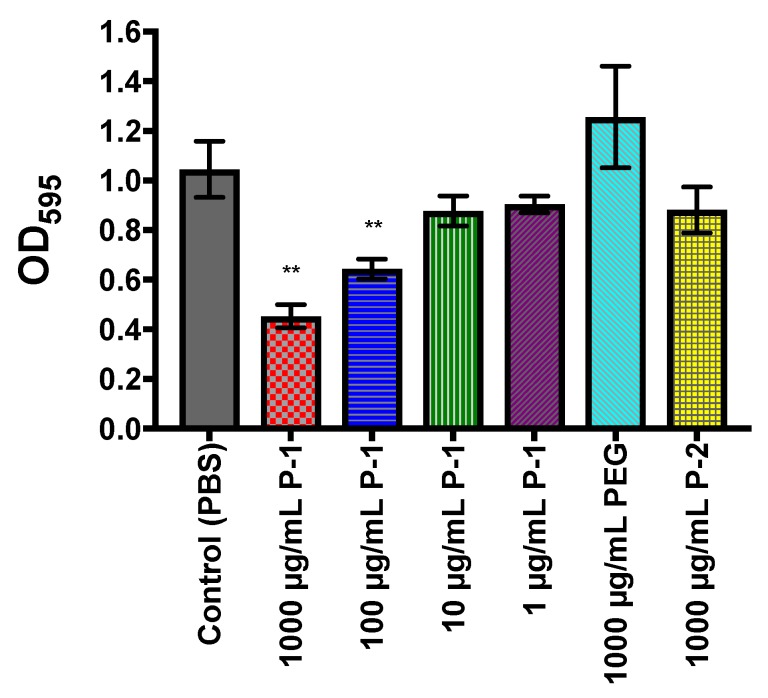
Crystal violet evaluation of polymer-modulated *P. aeruginosa* biofilm formation. Total biomass dependence on polymer concentration after 24 h of incubation in the presence of P-1, PEG, or P-2. PBS was used as a positive control. The experiment was performed in duplicate, and the absorbance was determined as the average of data for each experiment. The data points and error bars represent the average and s.d from data in three independent experiments (*n* = 3), with significance (** *p* ≤ 0.01) indicated against control (PBS).

We evaluated the total biomass of *P. aeruginosa* biofilm as a measure of biofilm formation to determine if cationic P-1 enhances or inhibits biofilm formation. Crystal violet (CV) staining has been an established method in biofilm microbiology to quantify the total biomass of biofilms owing to electrostatic binding of cationic CV to the anionic biopolymers of extracellular matrix and bacterial membranes in the biofilm [41,42]. The amount of CV adsorbed onto biofilms, extracted, and characterized by absorbance (OD_595_) reflects the total biomass of biofilms (Figure 7). 

In the presence of P-1, the total biomass as characterized by CV was decreased (Figure 7). This effect is concentration dependent, where biomass reduction is only evident at P-1 concentrations ≥100 µg/mL (Figure 7). The lack of change in biomass by PEG and P-2 suggest that the reduced biomass is the result of specifically cationic polymers. 

#### 2.4.2. Bacterial Biofilm Viability

**Figure 8:**
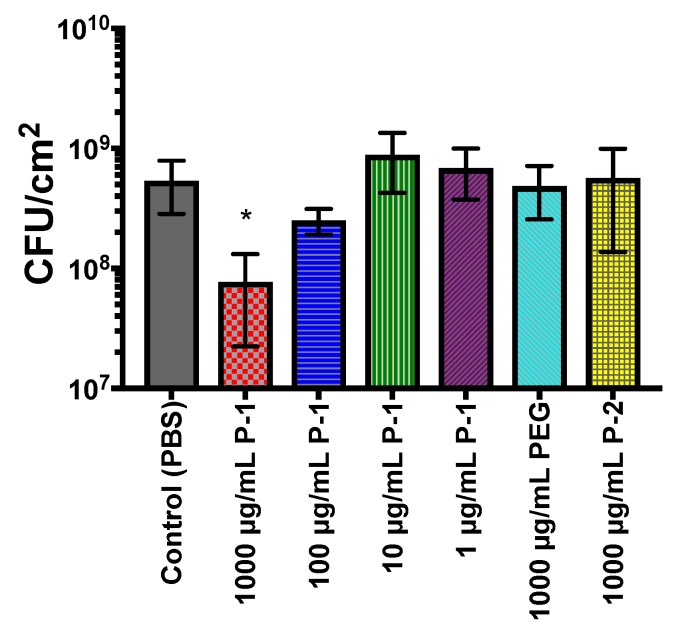
Viable bacteria evaluation of polymer-modulated biofilm formation. Viable bacteria dependence on polymer concentration after 24 h of incubation in the presence of P-1, PEG, or P-2. PBS was used as a positive control. The experiment was performed in duplicate, and the absorbance was determined as the average of data for each experiment. The data points and error bars represent the average and s.d from data in three independent experiments (*n* = 3), with significance (* *p* ≤ 0.05) indicated against control (PBS).

The biofilm formation was also quantified by the number of viable bacterial cells in the biofilms. When biofilms were formed in the presence of 1000 µg/mL P-1, the number of viable bacteria was reduced to 7.7 (±5.5) 10^7^ CFU/cm^2^ (Figure 8), which corresponds to an 86% reduction.

As P-1 concentration decreases however, the bacterial load approaches levels similar to the control. These results are in good agreement with findings based on the biomass by CV staining, presented in Figure 7. As P-1 has no antimicrobial activity, the reduction in viable cells is the result of inhibited biofilm growth. Incubation with PEG and P-2 did not significantly alter the viable bacteria load as compared to the control, with counts of 4.9 (±2.3) and 5.7 (±4.3) × 10^8^ CFU/cm^2^ respectively (Figure 8).

#### 2.4.3. Metabolic Activity of Bacterial Biofilms

**Figure 9:**
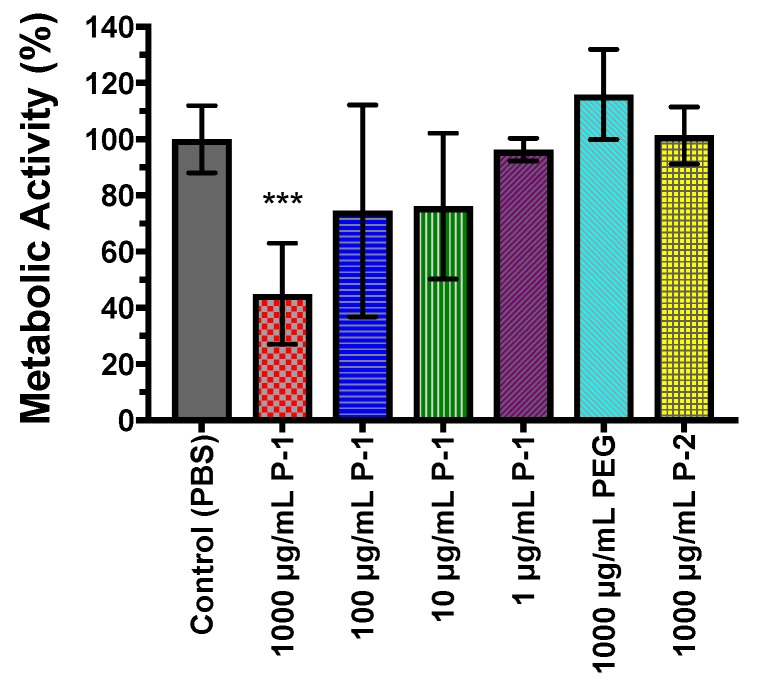
Metabolic activity assessed by MTT assay dependence on polymer concentration after 24 h of incubation in the presence of P-1, PEG, or P-2. PBS was used as a positive control. The experiment was performed in duplicate, and the absorbance was determined as the average of data for each experiment. The data points and error bars represent the average and s.d from data in three independent experiments (*n* = 3), with significance (*** *p* ≤ 0.001) indicated against control (PBS).

The viability of bacteria in biofilms was also investigate using cell metabolic MTT assay. *P. aeruginosa* was incubated with the polymers in a range of polymer concentrations for 24 h and allowed to form biofilms, and biofilms were subsequently assessed by MTT assay. Biofilms grown in the presence of PEG and P-2 demonstrated similar metabolic activity to control biofilms (Figure 9), which supports direct enumeration, and indicates bacteria are at similar metabolic states. 

The reduction of metabolic activity at 1000 µg/mL P-1 (Figure 9) is consistent with the lower number in viable bacteria found by direct enumeration. As P-1 concentration decreases, metabolic activity is increased to comparable levels to the control biofilms (Figure 9), supporting the finding of similar amounts of viable bacteria. These findings corroborate direct enumeration studies indicating bacterial reduction only in the presence of high concentrations of P-1, indicating cationic charge and sufficient charge is necessary to reduce biofilm accumulation. 

## 3. Discussion

In this study, we first investigated the antimicrobial activity of the polymers and their effect on the aggregation behavior of *P. aeruginosa*. The results indicated that P-1 did not inhibit the growth of planktonic *P. aeruginosa.* Some cationic polymers have been reported to exert bactericidal or bacteriostatic activity [43,44,45]. Typically, biocidal cationic polymers act by membrane disruption, where cationic segments bind onto the anionic bacterial surface, and hydrophobic sections of the polymer cause catastrophic defects in the membrane which release essential cellular components and ions. The lack of antimicrobial activity of P-1 may be explained by low molecular weight and absence of hydrophobic groups. This result also suggests that the suppression of biofilm development is not due to bacterial cell death. 

The flocculation data indicated that P-1 induced the aggregation of *P. aeruginosa*, but P-2 and PEG did not. In general, ionic polymers have been used to aggregate or flocculate charged molecules for applications such as waste water treatment or bioreactors [27,28,29]. Cationic polymers have been previously used to induce bacterial aggregation and flocculation by interacting with bacterial surfaces which have high net negative charge [46]. Electrostatic interactions between anionic bacteria surfaces and cationic polymers can result in surface neutralization, electrostatic patching and polymer bridging of bacterial cells, resulting in macroscopic aggregates or precipitates due to changes in repulsive and attractive forces [30,31,32]. The data suggested that P-1 at sufficient concentration can modulate the aggregation behavior of planktonic bacteria through electrostatic interactions. 

**Figure 10:**
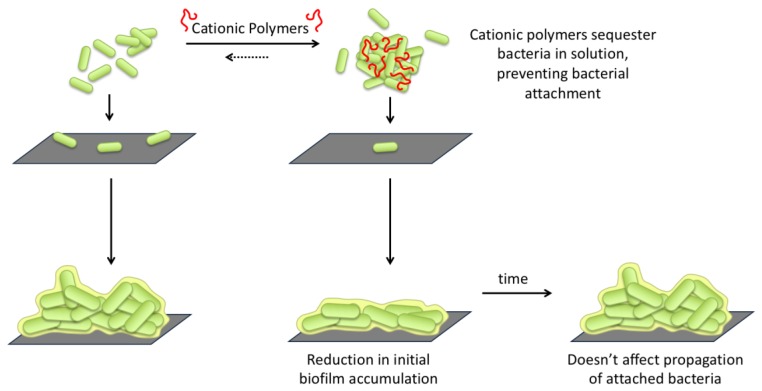
Proposed mechanism for cationic polymer modulation of biofilm behavior through planktonic bacteria interactions. Cationic polymers are proposed to sequester bacteria in solution, preventing bacterial attachment to surfaces, and consequentially suppression biofilm accumulation.

Based on the results described above, we propose a new mechanism of biofilm prevention through cationic polymer-bacteria interactions (Figure 10). 

In this mechanism, the observed aggregation of bacteria in the presence of cationic polymers results in bacteria sequestration in solution. The cationic polymers bind to the anionic surface of bacteria, which either results in charge neutralization which reduces repulsion between bacteria, or bridging between cationic and anionic portions of adjacent bacteria. This aggregation sequesters bacteria in solution, reducing the number of planktonic bacteria that can attach to a surface to initiate biofilm formation. Increasing the concentration of P-1 more effectively sequester the bacteria away from surfaces which they would otherwise attach, as results suggest in the reduction in biofilms. However, the polymer-bacteria interaction appears not to change the inherent ability of bacteria to attach to surface and form biofilms. Some bacteria eventually do attach, divide, and propagate as normal. P-1 can delay the biofilm formation. Studies that would further help elucidate the mechanism of action include identifying the relationship between polymer concentration, aggregate size, and degree of biofilm suppression. Additionally, further studies that monitor the accumulation of biomass and viable bacteria over time both during early development (2–12 h), and beyond 24 h would be useful to identify a point at which this approach no longer effectively combats biofilms, such as would be necessary for application in combination therapy studies. 

## 4. Materials and Methods

### 4.1. Materials

Tryptic soy broth (TSB, Thermo Scientific Oxoid™), Mueller Hinton broth (MHB, BD and Company^©^), and phosphate buffered saline (PBS, pH = 7.4, Gibco^®^) were prepared according to manufacturer instructions and autoclave sterilized prior to use. SYTO 9 and propidium iodide (PI) nucleic acid stains (Molecular Probes, OR) were used as prescribed by the product manual at 1.5 µL/mL. Crystal violet (Sigma-Aldrich) was diluted in Millipore water to a working concentration of 0.01% (*w*/*v*). MTT (3-(4,5-dimethylthiazol-2-yl)-2,5-diphenyltetrazolilum bromide, Thermo Scientific) was diluted in Millipore water to working concentration of 0.3% (*w*/*v*).

Polyethylene glycol (PEG, average M.W. = 6000 g/mol) and polyethylenimine (PEI, branched, approximate M.W. = 60,000 g/mol) were used as received from Acros Organics™. Poly[(3-methacryloylamino)propyl] trimethylammonium chloride (P-1) and poly(2-acrylamido-2-methylpropane sulfonic acid) (P-2) were synthesized by RAFT polymerization using a modified literature procedure and characterized by ^1^H NMR and GPC (Supplementary Material). Stock solutions were prepared at 20 mg/mL in 0.01% acetic acid). Further P-1 solutions were prepared by dilution in PBS.

*Pseudomonas aeruginosa* (*P. aeruginosa*) PAO1 was received from Dr. Chuanwu Xi (Environmental Health Science, University of Michigan).

### 4.2. Antimicrobial Activity Assessed by Minimum Inhibitory Concentration (MIC) Assay

The minimum inhibitory concentration (MIC) of polymers against *P. aeruginosa* (PAO1), *E. coli* (ATCC 25922), and *S. aureus* (ATCC 25923) were determined in a standard microbroth dilution assay according to the Clinical and Laboratory Standards Institute guidelines with suggested modifications by R. E.W Hancock Laboratory (University of British Columbia, British Columbia, Canada) [38] and Giacometti et al. [39]. *P. aeruginosa* was grown overnight (~18 h) in TSB at 37 °C with orbital shaking (180 rpm), and used as an inoculum by diluting overnight culture in TSB to a concentration of OD_600_ = 0.1. The inoculated solution was then grown at 37 °C to the exponential phase (OD_600_ = 0.5–0.7, 2 h). Final dilution to OD_600_ = 0.001, ~4 × 10^6^ CFU/mL, was made with TSB. Bacterial suspension (90 µL/well) was transferred to a 96-well sterile round-bottom polypropylene plate. Polymers were dissolved in 0.01% acetic acid to achieve stock concentrations of 20 mg/mL. Serial two-fold dilutions of polymers were prepared from stock solutions in PBS and transferred to the 96-well sterile round-bottom polypropylene plate for a final concentration of 7.8–1000 µg/mL (10 µL/well). PBS was used as a solvent control in place of polymer. Plates were sealed with parafilm and incubated for 18 h at 37 °C without shaking. MIC was defined as the lowest concentration of polymers to completely inhibit bacterial growth, as indicated by lack visual of turbidity. Assays were repeated a minimum of three times in triplicate on different days. The MIC of polymers against *P. aeruginosa, E. coli,* and *S. aureus* in MHB were assessed using the same protocol, with the substitution of TSB with MHB.

### 4.3. Bacterial Growth Curves by Optical Density

The effect of polymers on *P. aeruginosa* growth was assessed by optical density (OD_600_). Disposable polystyrene cuvettes were sterilized by 70% EtOH solution for and air dried. *P. aeruginosa* overnight cultures were diluted in TSB to desired concentration of OD_600_ = 0.001. A solution of bacterial suspension (900 µL) and stock polymer (P-1, P-2, or PEG, 100 µL) was prepared in cuvettes, and cuvettes secured with parafilm to prevent contamination. Final polymer concentrations were 1000 µg/mL or 100 µg/mL after dilution in the bacteria suspension. Solutions were incubated at 37 °C with orbital shaking (180 rpm) except when removed for time of measurement. The optical density (OD_600_) of dispersions were measured by optical density using a visible diode array spectrophotometer (WPA S800 Spectrawave, Biochrom). Measurements were taken at two and four hours, and then once an hour through 10 h, and a final measurement taken at 18 h. Triplicate measurements were carried out on separate days. 

### 4.4. Bacterial Aggregation/Flocculation Assessment by Optical Density

The effect of polymers on bacteria aggregation and/or flocculation was assessed by optical density (OD_600_), using sterilized cuvettes and bacteria as prepared in the previous section. A solution of bacterial suspension (900 µL) and stock polymer (P-1, P-2, PEG, or PEI, 100 µL) was prepared in cuvettes, and cuvettes secured with parafilm to prevent contamination. Final polymer concentrations were 1000 µg/mL or 100 µg/mL after dilution in the bacteria suspension. In contrast to growth curve assays, bacterial suspensions were kept at RT in the absence of shaking, to allow for bacteria to aggregate and precipitate in the absence of external forces. The optical density (O_D600_) of dispersions were measured by optical density using a visible diode array spectrophotometer (WPA S800 Spectrawave, Biochrom). An OD_600_ reading was taken prior to polymer addition (time = −0.05 min), and immediately following polymer addition (time = 0 min). Measurements were taken at 0, 5, and 10 min, subsequently every 10 min for the first hour and every 20 min during the second hour. To prevent agitation of the bacterial suspension, cuvettes remained in the spectrophotometer for the entirety of the measurement time. Triplicate measurements were carried out on separate days. 

### 4.5. Bacterial Aggregate Observation: Confocal Microscopy

*P. aeruginosa* overnight cultures were diluted in TSB to desired concentration of OD_600_ = 1.0. Bacteria suspensions (900 µL) were combined with stock polymer solutions or PBS (100 µL) to achieve final polymer concentrations (1000–1 µg/mL) in an Eppendorf tube. SYTO 9 (1.5 µL) was added and incubated with suspensions for 15 min at RT in the absence of light without shaking, simultaneously allowing time for polymer-bacteria interaction. An aliquot (5 µL) was collected from the bottom of Eppendorf tube and deposited on glass microscope slides and covered with glass coverslips, with the manual removal of air bubbles. Bacteria was examined by inverted confocal microscope (Eclipse Ti-U, Nikon C2 Plus) using a Plan Apo VC 60× Oil DIC N2 objective. Laser excitations were 488.0 (SYTO9, observation: 525.0 nm) at power 4.0, observed with a pinhole size of 30.0 µm and scan speed of 0.125. 

### 4.6. Bacterial Formation Over Time: Confocal Microscopy

*P. aeruginosa* overnight cultures were diluted in TSB to desired concentration of OD_600_ = 0.001. Bacterial suspensions (2.7 mL) were combined with P-1 solutions (0.3 mL) or PBS (0.3 mL) to achieve desired P-1 concentration (1000 µg/mL). The prepared bacteria-polymer solutions were transferred to a clear cell culture dish (FD35-100, Fluorodish™) with glass window diameter of 23.5 mm, sealed with parafilm, and incubated for two, four, or eight hours at 37 °C in the absence of shaking. Following incubation, the culture medium was carefully removed from the wells by micropipette to avoid disrupting biofilms. Wells were washed twice with an excess of PBS. Biofilms were stained by SYTO 9 (3.34 mM) and/or propidium iodide (20 mM) in 0.85% NaCl solution for 20 min at RT in the absence of light without shaking. SYTO 9-PI solution was then removed by micropipette and biofilms washed once with 0.85% NaCl solution. Biofilms were examined by inverted laser scanning confocal microscope (Eclipse Ti-U, Nikon C2 Plus) using a Plan Apo VC 60× Oil DIC N2 objective. Laser excitations were 488.0 (SYTO9, observation: 525.0 nm) and 561.0 nm (propidium iodide, observation: 600.0 nm), both at power 4.0, observed with a pinhole size of 30.0 µm and scan speed of 0.125. Automated Z-stacked images were acquired with 1 µm step distance, and rendered in the Nikon NIS-Elements AR software without deconvolution.

### 4.7. Biofilm Formation with Polymer Co-incubation

Biofilm growth was performed with modifications to previous literature methods [47,48]. 12 mm diameter borosilicate glass coverslips (Fisherbrand™) were sterilized in 70 vol% ethanol and allowed to air dry. Label tape was affixed by the self-adhesive to the bottom of wells in a sterile 12-well polystyrene plates (Corning^®^ Costar^®^), and the plate sterilized with 70 vol% ethanol and allowed to dry completely. Coverslips were placed 1 per well, with the entire coverslip being flush with the well bottom. The purpose of the label tape was to provide a method to disrupt the capillary action between the well bottom and coverslip, therefore allowing a fixed area of biofilm (adhered to the coverslip) to be removed for analysis with minimal disruption. *P. aeruginosa* overnight cultures were diluted in TSB to desired concentration of OD_600_ = 0.001 Bacteria solution (900 µL) was added to each well to fully immerse the coverslip. Polymer stock solutions or PBS (100 µL) were then added to wells to achieve desired polymer concentrations (1000–1 µg/mL). TSB with PBS (100 µL) was used as controls to confirm lack of bacterial contamination. Plates were wrapped in parafilm and incubated for 24 h at 37 °C in the absence of shaking.

### 4.8. Evaluation of Total Biomass by Crystal Violet Staining Assay

Biofilms were generated as described in the previous section. Following incubation, the supernatant was removed from the wells by micropipette. Wells were washed twice with excess PBS. Crystal violet (0.01 *w/v*%, 1000 µL, CV) solution was introduced to wells by micropipette and used to stain the biofilm for 15 min at RT without shaking. CV was removed from the wells by micropipette, and wells were washed twice with PBS. In order to maintain a consistent biofilm area, coverslips (with adhered biofilms) were removed and transferred to a new sterile 12-well plate using forceps, by holding a coverslip edge carefully as to not disrupt the biofilm. Ethanol (100%, 1000 µL) was used to extract CV from the biofilms for 10 min at RT and the biofilm manually disrupted by micropipette agitation of the solution and scraping. An aliquot of the solution (200 µL) was transferred to 96-well round-bottom plate, which was centrifuged at 1000 rmp for 5 min in order to reduce the impact of cellular debris in solution. Aliquots (25 µL) of solution were then transferred to untreated, sterile polystyrene 96-well flat-bottom plate containing ethanol (100%, 75 µL) to create 1:4 solutions and the OD_595_ was read on a Varioskan Flash microplate reader (Thermo Fisher). Experiments were carried out in duplicate on three separate days. Daily averages were calculated from duplicates and used to determine a grand average and standard error of the mean (*n* = 3).

### 4.9. Evaluation of Viable Bacteria by Direct Enumeration 

Biofilms were generated as described in the previous section. Following incubation, the supernatant was removed from the wells by micropipette. Wells were washed twice with excess PBS. Washed coverslips (with adhered biofilms) were removed and transferred to 15 mL conical tubes containing PBS (3 mL) using forceps, by holding a coverslip edge carefully as to not disrupt the biofilm. Coverslips in PBS were sonicated for 10 min to fully disrupt biofilms and dispersed bacteria in solution. 10-fold serial dilutions were performed in PBS, and an aliquot of samples (100 µL) was plated on TSB agar plates. The agar plates were incubated overnight (~18 h) at 37 °C, and the number of viable colonies was determined. An appropriate dilution was selected from agar plate contained 30–300 colonies, the accepted range of countable colonies. Experiments were carried out in duplicate on three separate days. Daily averages were calculated from duplicates and used to determine a grand average and standard error of the mean (*n* = 3). 

### 4.10. Evaluation of Metabolic Activity by MTT Assay

Biofilms were generated as described in the previous section. Following incubation, the supernatant was removed from the wells by micropipette. Wells were washed twice with excess PBS. To each well, a premixed solution of PBS (750 µL) and 0.3% MMT (250 µL) were added and allowed to incubate for two hours at 37 °C in the absence of stirring. The solution was removed by micropipette and the wells washed with PBS, and the solution discarded. Washed coverslips were removed and transferred to a new 12-well plate using forceps, by holding a coverslip edge carefully as to not disrupt the biofilm. A solution of DMSO (750 µL) and glycine buffer solution (pH 10.5, 126 µL) was added to each well with coverslip and the biofilms manually disrupted by solution agitation and scraping and allowed to incubate at RT for 15 min. Aliquots (100 µL) of solution were then transferred to untreated, sterile polystyrene 96-well-plate and the OD_570_ was obtained on a Varioskan Flash microplate reader (Thermo Fisher). Experiments were carried out in duplicate on three separate days. Daily averages were calculated from duplicates and used to determine a grand average and standard error of the mean (*n* = 3).

### 4.11. Statistical Analysis

The assays were performed in duplicate or triplicate in three independent experiments in different days. In the case of biofilm experiments, duplicate data in the same day was averaged to give a daily average. The daily average values from three independent experiments were used to calculate the average value and standard deviation. The data were analyzed by Student’s *t*-test. Statistical significance was determined as * *p* ≤ 0.05, ** *p* ≤ 0.01, or *** *p* ≤ 0.001. 

## 5. Conclusions

In conclusion, cationic poly[(3-methacryloylamino)propyl] trimethylammonium chloride (P-1) is capable of modulating planktonic and biofilm *P. aeruginosa* behavior through electrostatic interactions. Specifically, P-1 has been shown to induce concentration dependent bacteria aggregation in solution, indicating changes to the physiochemical surface of bacterial membranes. Despite no direct antimicrobial action, P-1 suppressed biofilm accumulation of both matrix and viable bacteria when concentrations ≥100 µg/mL were incubated with planktonic bacteria during biofilm development, while neutral and anionic polymers did not. We propose that cationic polymers can sequester planktonic bacteria in solution through electrostatic interactions, preventing bacteria attachment and consequently reducing biofilm formation. This approach could be useful in combination with existing antibiotics to reduce the necessary antibiotic dose in clinical applications by suppressing biofilm development at early stages. Our findings provide a mechanistic link between the polymer-bacteria interactions and biofilm formation, useful to develop versatile anti-biofilm polymers which may modulate not only bacteria-material interactions, but also interfere with bacteria-host interactions for infection prevention. 

For the future perspective, in order to advance this approach towards clinical applications and increase our knowledge on the functional role of cationic polymers in biofilm formation, the toxicity of the polymer to human cells should be considered and evaluated. We also believe that this approach is not unique to P-1, but also other cationic polymer structures can be used to achieve control of biofilm formation. This is because the proposed mechanism relies on the physicochemical (electrostatic) interactions between bacteria and polymers, but not biological effects, such as bactericidal effects or signal-promoted biofilm dispersion. While we used only one model polymer to test the hypothesis, the ability of polymers to sequester bacteria is likely to dependent on polymer length, charge density, and chemical types of ammonium groups (primary, ternary, etc.), which govern the binding of polymer chains to bacterial cell surfaces and the bridging of bacterial cells. For future work, cationic polymers with different chemical compositions, polymer lengths, and polymer structures can be studied for optimization for their potent anti-biofilm efficacy and validation of the proposed mechanism. 

## Figures and Tables

**Table 1:** Polymer characterization of P-1 and P-2.

Polymer	DP	*M*_n_ (g/mol) (^1^H NMR)	*M*_n_ (g/mol) (GPC)	*M*_w_ (g/mol) (GPC)	*Đ*
P-1	116	25,900	19,600	20,580	1.05
P-2	99	20,589	15,333	20,392	1.33

**Table 2:** Antimicrobial activity of P-1, P-2, and PEG.

Polymers	MIC (µg/mL)		
	*P. aeruginos ^a)^*	*E. coli ^b)^*	*S. aureus ^b)^*
P-1	>1000	>1000	>1000
P-2	>1000	>1000	>1000
PEG	>1000	>1000	>1000

a) Determined using tryptic soy broth (TSB) and Mueller Hinton broth (MHB); b) determined using MHB.

## Supplementary Materials

The following are available online at https://www.mdpi.com/2079-6382/8/2/61/s1; **Figure S1.** ^1^H NMR spectra for (**a**) P-1 and (**b**) P-2 in D_2_O; **Figure S2.** GPC elution curves for (**a**) P-1 using two Shodex Ohpak SB-804 HQ columns and a 0.3 M Na_2_SO_4_ aqueous solution containing 0.5 M acetic acid as an eluent and (**b**) P-2 using a Shodex Asahipak GF-7M HQ column and a phosphate buffer (pH 9) containing 10 vol% acetonitrile as an eluent.
