# Supplementary material for: Solution-Mediated Modulation of Pseudomonas aeruginosa Biofilm Formation by a Cationic Synthetic Polymer
Source: Antibiotics (Basel). 2019 May 10;8(2):61. doi: 10.3390/antibiotics8020061 (PMC6627362; doi:10.3390/antibiotics8020061)
Supplement: Supplementary file 1 [file antibiotics-08-00061-s001.pdf]

Supplementary Material

Solution-Mediated Modulation of *P. aeruginosa* Biofilm Formation by a Cationic Synthetic Polymer

Leanna Foster [1], Shin-ichi Yusa [2] and Kenichi Kuroda[1,3,*]

[1] Macromolecular Science and Engineering, University of Michigan, Ann Arbor, MI, 48109; lllfoster@umich.edu
[2] Department of Materials Science and Chemistry, University of Hyogo, 2167 Shosha, Himeji, Hyogo 671-2280, Japan; yusa@eng.u-hyogo.ac.jp
[3] Department of Biologic and Materials Sciences & Prosthodontics, School of Dentistry, University of Michigan, Ann Arbor, MI 48109; kkuroda@umich.edu

* Correspondence: kkuroda@umich.edu

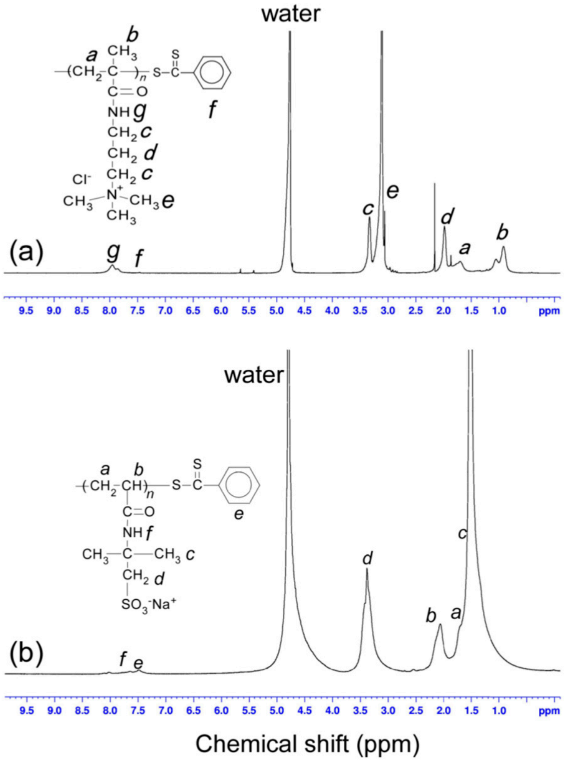

Figure S1. ^{1}H NMR spectra for (a) P-1 and (b) P-2 in D_2O. The ^{1}H NMR spectra were obtained with Bruker DRX-500.

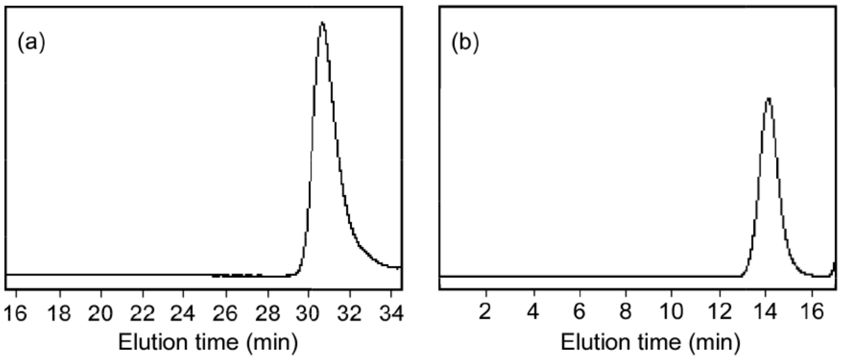

Figure S2. GPC elution curves for (a) P-1 using two Shodex Ohpak SB-804 HQ columns and a 0.3 M Na_2SO_4 aqueous solution containing 0.5 M acetic acid as an eluent and (b) P-2 using a Shodex Asahipak GF-7M HQ column and a phosphate buffer (pH 9) containing 10 vol% acetonitrile as an eluent.
